# Does 3D Ultrasound Enhance the Diagnosis of Bladder Tumours in Patients with Haematuria?

**DOI:** 10.5402/2012/158437

**Published:** 2012-03-07

**Authors:** Miguel Silva-Ramos, Nuno Louro, Rui Versos, Victor Cavadas, Filinto Marcelo

**Affiliations:** Department of Urology, Hospital Geral de Santo António, CHP, 4099-001 Porto, Portugal

## Abstract

*Purpose*. Bladder cancer is a frequent cause of haematuria in elderly patients, and bladder ultrasound (US) is a valuable tool in diagnosing these malignancies. We examined the accuracy of 3D bladder US in diagnosing bladder tumors in patients with haematuria. *Patients and Methods*. Twenty-one patients observed in the emergency department for haematuria underwent a kidney and bladder US. Patients with normal or uncertain bladder US findings underwent a 3D US and a cystoscopy. *Results*. In 5 (23.8%) patients, the 3D US detected bladder tumours not seen in 2D US. All these patients were found to have bladder tumours on cystoscopy. Another 5 (23.8%) patients with uncertain findings on 2D US had normal 3D US and cystoscopy. 3D US showed a sensitivity of 83.3% and a specificity of 100% with a positive predicted value and negative predictive values of 100% and 93.8%, respectively. *Conclusion*. 3D US was more sensitive than 2D US in diagnosing bladder tumours in patients with haematuria.

## 1. Introduction

Gross haematuria can be a very distressing and frightening symptom and usually leads patients to seek immediate medical care. The cause of haematuria should be thoroughly evaluated and in adults should be regarded as a sign of malignancy until proven otherwise. Ultrasound (US) is one of the initial examinations performed and is very useful in detecting kidney and bladder lesions.

The sensitivity of US in detecting bladder tumours (BT) is variably reported to range from 26% to over 80% [1, 2]. However, this rate is much lower in patients with tumours smaller than 5 mm or for tumours located on the bladder dome or anterior wall [2]. Similarly, specificity is decreased in the presence of coexisting focal cystitis, bladder trabeculations, and haematuria with clots. For this reason, a significant number of bladder US are normal or inconclusive, and cystoscopy is necessary to discard the presence of a bladder tumour [3].

Three-dimension US imaging has recently become a widely available feature in many ultrasound machines. This technology permits the acquisition and storage of a dataset, selected from a specific region of interest. This dataset can be further analysed, either by multiplanar display, surface rendering, or volume calculation. As there is a considerable contrast gradient between the bladder lumen and its wall, the surface rendering algorithm can usually display with sufficient detail the surface of the bladder, revealing a cystoscopic-like image, enhancing the characterization of bladder wall abnormalities [4].

In this study, we examined the value of 3D US in diagnosing bladder tumours in patients with haematuria of unknown cause.

## 2. Patients and Methods

Patients admitted in the emergency department presenting with painless gross haematuria and without history of trauma or evidence of urinary tract infection were prospectively enrolled. All patients underwent conventional 2D bladder and kidney US in the emergency department performed by a radiologist. When needed, bladder clot evacuation was performed previous to the US. Examining physicians were asked to report the bladder US as “with BT” or “no BT.” In some patients, the radiologist observed bladder lesions that were more difficult to characterize, because of unclear limits or image artefacts. These cases were reported as “uncertain.” Patients diagnosed with kidney disease, bladder tumours, or calculi causing the haematuria were excluded. Patients with “no BT” or “uncertain” bladder US findings were scheduled for a 3D US examination, usually the next day, followed by flexible or rigid cystoscopy.

A Philips HDI 4000 ultrasound system (Philips Medical Systems, Best; Netherlands) with a 3.5–5 MHz transabdominal probe was used. A single urologist performed the 3D US examination. Patients were asked to drink about 1 L of water and not to void 1 h before the study in order to perform it with near maximum bladder capacity (volume ≥ 250 cc), a 2D US was carried out in the sagital and transverse plane, and afterwards several 3D acquisitions (2 to 5 datasets, mean 2,5) were made and evaluated using the surface rendering algorithm.

Comparative analysis was carried out between the imaging findings collected by 2D and 3D US to those of the cystoscopy. Cochrane's *Q* test was used to determine statistical differences.

## 3. Results

Twenty one patients (19 men and 2 women, mean age 69.0 years old) were enrolled in the study. Twelve (57%) where reported as “no BT” and 9 (43%) as “uncertain” on the conventional 2D US performed in the emergency department.

**Figure 1:**
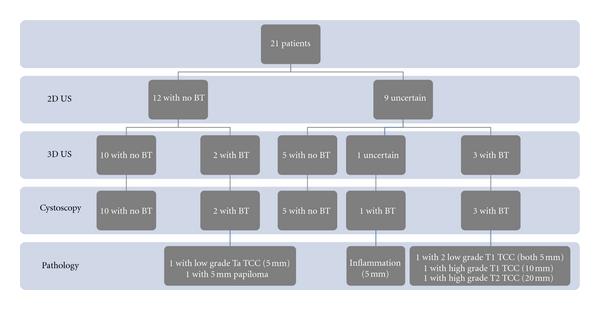
Results of 2D US, 3D US, cystoscopy, and pathology examination.

The 3D examination found “no BT” in 15 patients (71.4%). In 5 of these patients, the 2D US was “uncertain,” largely because they had bladder trabeculation that made the 2D US difficult to interpret. None of these patients had bladder tumour on cystoscopy (Figure 1).

In 5 (23.8%) patients, 3D US identified bladder tumours that were not detected in the previous US examination. This was statistically different from the results of 2D US (*P* = 0,025). All these patients were found to have bladder tumours on cystoscopy, although in one patient cystoscopy revealed another 5 mm lesion that was not detected on US.

In one patient the 3D US was inconclusive, but followup cystoscopy revealed a bladder tumour.

All 6 patients with bladder tumours on cystoscopy underwent a transurethral resection; 4 had transitional cell carcinomas (TCCs), 1 had a papilloma, and the remaining patient was revealed to have only inflammation. Lesion diameter ranged from 5 to 20 mm (mean 7 mm).

In the population with negative 2D US, the 3D US showed a sensitivity of 83,3% and a specificity of 100% in identifying patients with BT, with a positive predictive value and negative predictive values of 100% and 93,8%, respectively.

## 4. Discussion

 When evaluating patients with painless haematuria, it is mandatory to exclude the existence of tumours of the urinary tract. US imaging is one of the frontline examinations since it is widely available and noninvasive. When a US clearly shows a bladder tumour, a transurethral resection is usually subsequently performed; however, when the US examination is normal or ambiguous, a diagnostic cystoscopy becomes mandatory. Flexible cystoscopy, though less traumatic, is still an invasive procedure associated with patient discomfort and can by itself be the cause of urinary tract infections and haematuria. In order to overcome these problems, other imaging techniques such as contrast enhanced US [8, 9], and virtual cystoscopy performed with computed tomography or magnetic resonance imaging, have been developed with promising results [10–13]. These techniques seem to be more sensitive than US but significantly more time consuming, expensive, and frequently inaccessible for clinicians. Moreover, all these imaging techniques are unable to detect subtle alterations of bladder mucosa such as carcinoma in situ [13]. For such lesions, cystoscopy and, more recently, fluorescence cystoscopy and narrow-band imaging cystoscopy are still the most accurate examinations [14, 15].

Three-dimensional ultrasound is now an established imaging tool in several specialities, this technology is available in most medium and upscale equipments and is routinely used in obstetric US. In urology, it has been used in planning and guiding prostate cancer treatments [16], to accurately measure bladder volume [17] and in imaging the urethral sphincter in pelvic floor disorders [18]. Recently, the use of 3D US in the evaluation of bladder tumours has been reported showing a moderate-to-perfect interobserver agreement [6]. Wagner et al. [19] studied 3D US as a staging tool in distinguishing invasive from noninvasive bladder tumours, showing an overall accuracy of 79%. This technology has also been studied as a screening tool; Mitterberger et al. [5] studied 42 patients with painless haematuria, all of whom had an abnormal 2D US. They found that 3D US diagnosis was correct in 86% of patients whereas 2D US diagnosis was correct only in half of them. Another group [7] compared the accuracy of 2D and 3D US in 14 patients with proven BT and found 3D US to have better sensitivity. In our study, we evaluated only patients with an undetermined cause of haematuria after 2D US, and we similarly found greater sensitivity of 3D US in detecting bladder tumours (Table 1). However, this sensitivity was not related to lesion size since 57% of lesions were 10 mm or more. This might be explained by the fact that the 3D reconstruction facilitates the distinction of tumour lesions from bladder folds, becoming particularly useful in patients with bladder trabeculations and diverticula. Although cystoscopy is still mandatory in those patients with an unidentified cause of haematuria, we think that 3D US can avoid cystoscopies by clearly identifying and accurately characterizing bladder lesions. In this series, 5 patients (23.8%) could have skipped cystoscopy and be directly referred for a transurethral resection, saving patient discomfort, time, and money.

Although not prospectively assessed in this study, the specificity of 3D US seems to be superior to 2D US. In our series, in 5 patients which had uncertain findings on 2D US, the subsequent 3D reconstruction has shown better characterization of their lesions and clearly identified bladder trabeculae and prostatic median lobe that could mimic a BT on 2D US. Further investigation is necessary to determine the actual improvement in specificity of 3D US in these patients.

We are aware that in this study 2D and 3D US were performed under different conditions: the first was done in the emergency department by a radiologist, and the second in a different day by a urologist. The degree of haematuria may also have been different between the two examinations, however, clot removal was performed before the US whenever necessary, so the degree of haematuria should not have a major impact on the exam performance.

**Figure 2:**
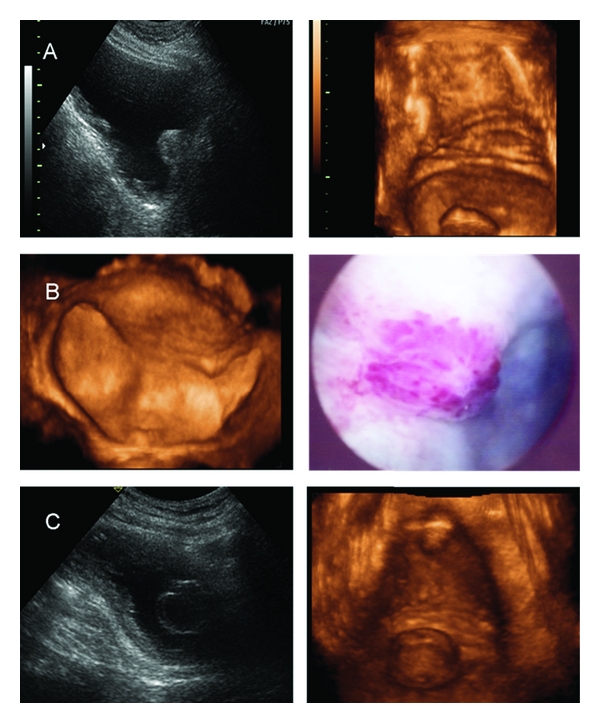
(a) View of a trabeculated bladder and prostatic median lobe in 2D and 3D US. (b) View of a small bladder tumour (papilloma) in 3D US and cystoscopy. (c) 2D and 3D view of a bladder dome tumour in a patient with an indwelling catheter.

Bladder 3D US is simple to perform and interpret. We usually carry out 2 to 3 volume acquisitions with a mechanical motor-driven transducer and evaluate the images using a surface-rendering algorithm. The images generated are clearly identified as the bladder lumen (Figure 2). Bladder tumours appear as round or oval protrusions into the bladder lumen and most software allows navigation through the surface of the bladder, and looking at the lesions from different angles. Overall, this procedure may add as little as 5 to 10 minutes to the examination time but substantially improves the overall accuracy of the exam, therefore, we think that this technology when available should be used in the diagnostic workup of patients with haematuria of unknown cause.

## 5. Conclusion

Three-dimensional bladder US has superior sensitivity than 2D US in diagnosing bladder tumours in patients with haematuria. Although cystoscopy is still the gold standard examination, 3D US is a promising tool in diagnosing bladder tumours and might reduce the number of cystoscopies needed to study these patients.

## Figures and Tables

**Table 1:** Overview of published studies on 3D bladder ultrasound in the diagnosis of bladder tumours.

Reference	Number of patients	Number of tumour lesions detected		
		2D	3D	Cystoscopy
Mitterberger et al. [5]	42	29	37	37
Kocakoc et al. [6]	28	39	41	47
Park et al. [7]	14	19	22	28
Current study	21	0	5	7

Total	105	87	105	119
Sensitivity* (%)		73,1	88,2	100

*Calculations were made considering cystoscopy as the “gold standard”.
